# A Monitoring Method Based on FDALM and Its Application in the Sintering Process of Ternary Cathode Material

**DOI:** 10.3390/s22197203

**Published:** 2022-09-22

**Authors:** Ning Chen, Fuhai Hu, Jiayao Chen, Kai Wang, Chunhua Yang, Weihua Gui

**Affiliations:** School of Automation, Central South University, Changsha 410083, China

**Keywords:** multimodality, factor modeling, process monitoring, FDALM

## Abstract

In industrial processes, the composition of raw material and the production environment are complex and changeable, which makes the production process have multiple steady states. In this situation, it is difficult for the traditional single-mode monitoring methods to accurately detect the process abnormalities. To this end, a multimode monitoring method based on the factor dynamic autoregressive hidden variable model (FDALM) for industrial processes is proposed in this paper. First, an improved affine propagation clustering algorithm to learn the model modal factors is adopted, and the FDALM is constructed by combining multiple high-order hidden state Markov chains through the factor modeling technology. Secondly, a fusion algorithm based on Bayesian filtering, smoothing, and expectation-maximization is adopted to identify model parameters. The Lagrange multiplier formula is additionally constructed to update the factor coefficients by using the factor constraints in the solving. Moreover, the online Bayesian inference is adopted to fuse the information of different factor modes and obtain the fault posterior probability, which can improve the overall monitoring effect of the model. Finally, the proposed method is applied in the sintering process of ternary cathode material. The results show that the fault detection rate and false alarm rate of this method are improved obviously compared with the traditional methods.

## 1. Introduction

Process monitoring plays an important role in ensuring product safety, improving product quality, and reducing production costs. Therefore, it is an important way to ensure the safe and efficient operation of the production process [1,2,3]. Multivariate statistical process monitoring (MSPM) has become increasingly popular with the proliferation of digital factories and the massive collection of production process data. The traditional representative techniques include Principal Component Analysis (PCA) and Partial Least Squares (PLS), which are used to monitor chemical production processes and achieve results [4,5,6].

However, the dynamics information contained in sequence data has been greatly ignored by most process monitoring [7]. Scholars have proposed dynamic augmented matrix [8], autoregressive [9], and linear dynamic systems [10] to monitor the dynamic process. However, the augmented matrix increases the curse of dimensionality. Moreover, the order of the autoregressive model and the dimension of the latent state is difficult to determine, resulting in limited monitoring performance [11]. Fortunately, linear dynamic systems, as a dynamic latent variable model, are good at describing the interaction between first-order dynamic process states. Aiming at the high-order dynamic process [12], Chen et al. [13] proposed a monitoring method of the dynamic autoregressive latent variable model to monitor the preparation process of ternary cathode material. However, the model assumes that the system operates stably under a single operating condition and fails for multimodal processes [14].

Researchers have made several contributions to addressing the multimodal problem [15,16]. A traditional approach is to build different monitoring models for various operating modes. Zhao et al. [17] proposed a mixed principal component analysis model for monitoring multimodal processes. It established PCA models for multiple data sets to distinguish different modal information. At the same time, Zhao et al. [18,19] proposed methods of PCA and multiple PLS models to handle multimodal processes. This type of method has several drawbacks: not fusing the correlation information between different models; splitting the useful information hidden between the data sequences; and being highly dependent on the similarity measurement algorithm. To address such problems, Bayesian frameworks and combinatorial strategies have been adopted. Yu et al. [20,21] proposed a monitoring method for the Gaussian mixture model and used the Bayesian inference method to fuse various model indicators. Ge et al. [22] proposed a hybrid PPCA model combined with Bayesian inference technology for process monitoring. The above methods attempt to construct a unified mathematical model, and then use Bayesian inference techniques to fuse the difference information between the models. However, they do not consider the information between the data sequences in the transition period. To this end, Wang et al. [23] proposed a Hidden Markov Model-based fault monitoring method for multi-mode processes, which relies on the accuracy of the partitioning algorithm for steady-state and transition-state data sets.

The multi-mode process monitoring method proposed above highly depends on the similarity measurement algorithm to categorize the data samples into a certain group, while ignoring the state action relationship between sequences. Due to the above reasons, the samples during the mode switching cannot be effectively considered, which may increase the error detection rate caused by misclassification [24]. To make full use of the information between sequences and preserve the interaction between multimodal states, a multimodal process monitoring method based on the factor dynamic autoregressive latent variable model is proposed in this paper. First, from a data perspective, a factor dynamic autoregressive latent variable model, which is obtained by combining multiple high-order hidden state Markov chains through factor modeling technology, is constructed. Then, the state output of the sample in each mode is fused to the posterior failure probability of the sample through Bayesian inference online, so as to take into the overall monitoring effect of the multimodal model. The main contributions can be summarized as: (1) with the help of factor modeling technology, multiple high-order Markov state chains are combined to obtain FDALM to simulate the dynamic evolution of multimodal processes; (2) a parameter identification algorithm is proposed to learn the FDALM parameters; (3) Bayesian technology is used to fuse different sub-modal features into sample posterior failure probability to achieve process monitoring tasks and reduce the false alarm rate; (4) focuses on solving the multimodal problems in the sintering process of ternary cathode materials. The proposed method is applied to the sintering process of ternary cathode material manufacturing. The results have practical guiding significance.

The main structure of the paper is arranged as follows. Section 2 introduces the difficulties in monitoring the preparation process of ternary cathode material. Section 3 presents the modeling method of the factor dynamic autoregressive latent variable model and introduces the parameter identification algorithm of the model in detail. A monitoring method based on FDALM is proposed in Section 4. Section 5 applies the monitoring method to monitor the sintering process of ternary cathode material to verify the effectiveness of the proposed method. The last section provides the conclusion.

## 2. Description of the Problem in Monitoring the Preparation Process of Ternary Cathode Material

**Figure 1:**
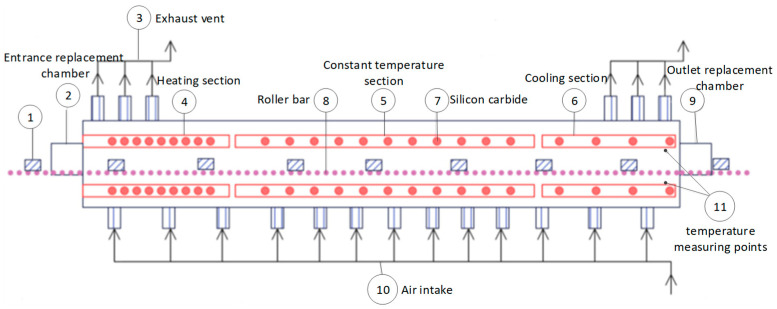
Sectional view of a roller kiln.

The roller kiln, which has a total length of more than 40 m and consists of 21 temperature zones, is a device used for the sintering reaction of ternary cathode material. It is divided into three temperature sections according to the temperature trend: the heating section, the constant temperature section, and the cooling section. Each temperature section is spliced together by several temperature zones, and its cross-sectional view is shown in Figure 1. The material is loaded into the saggar, enters the replacement chamber at the entrance of the roller kiln, and starts a series of chemical reactions. The qualified product relies on the temperature of the internal temperature zone of the roller kiln, the transmission power, and the supply of oxygen. The heating system adjusts the temperature field distribution in the temperature zone by controlling the current flowing on the silicon carbide rod, so as to ensure the uniform heat conduction of the material during the transmission process. The transmission system is driven by the rollers moving at a constant speed to help the saggars placed on the rollers to move forward smoothly and slowly at a certain speed. Oxygen is introduced into the air inlet to provide oxygen for the compound reaction, and the exhaust gas is extracted from the reaction. This system further ensures the stable air pressure condition in the roller kiln. In the ternary cathode material roller kiln, the temperature is mainly measured by thermocouples. Two thermocouples are at the top and bottom of each temperature zone, close to the upper and lower silicon carbon rods.

Due to the limitation of the production environment, there is a certain hysteresis relationship between the control parameters and the sintering state, so it is impossible to directly evaluate the quality of the production and preparation process by controlling variables such as silicon carbide rod current and oxygen concentration. At the same time, the quality of the ternary material is related to the temperature of the entire process, and the collected temperature variables have high dimensions, so it is difficult to measure whether the process state is in a normal state through a simple weighted average index. In addition, in order to compensate for the influence of environmental factors, there is more than one optimal sintering system for the sintering preparation process [25]. For example, the operating parameters in winter and summer are different, so a single evaluation criterion cannot be applied to the preparation process of ternary material. These problems make it difficult for operators to accurately grasp the state of on-site sintering preparation in real-time, resulting in side effects such as lag in operation and fluctuations in product quality. Therefore, it is very important to study the process monitoring technology to fully analyze the data collected by the process sensor to guide the operator to grasp and adjust the operating parameters in time, so that the process can return to its normal state.

**Figure 2:**
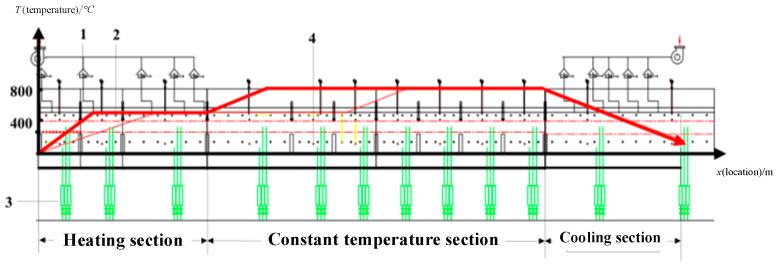
Variation trend of the internal temperature of a roller kiln.

That is to say, a qualified battery product requires that the distribution of the internal temperature field of the roller kiln conforms to a certain curve, that is, the temperature trend is shown in Figure 2. In Figure 2, 1 is the furnace exhaust port, 2 is the upper silicon-carbon rod, 3 is the furnace air inlet, and 4 is the lower heated silicon-carbon rod. Since the process conditions are directly determined by the sintering temperature, the temperatures in every section and product quality need to be modeled and monitored. The product quality is related to the temperatures of the entire section, and the front and rear temperature zones affect each other, so the monitoring of a single temperature zone cannot achieve the overall monitoring goal. However, thermocouples are installed at the top and bottom of each temperature zone inside the sintering device as temperature measuring elements to measure the temperature of the kiln. These temperatures can indirectly determine the quality of the products in the current kiln as a whole. At the same time, to monitor the faults related to product quality, such as the abnormal increase of residual lithium in the outlet replacement chamber, it is necessary to increase the monitoring of product quality based on monitoring temperature variables. The indicator that can best reflect product performance is surface free radicals. When the free lithium content increases, the material easily absorbs carbon dioxide and moisture in the air to generate lithium carbonate and lithium hydroxide, which not only reduces the storage stability of the material in the air but also hinders the deintercalation and electron conduction of lithium ions in the raw material.

The curve in Figure 2 records the temperature distribution inside the kiln that needs to be achieved under the production of qualified products. The actual kiln temperature is affected by the external environment, and the controller will eliminate these effects to adjust the temperature profile inside the kiln. Therefore, the sintering regime that deviates from the curve in Figure 2 is also consistent with the normal production process and cannot be judged as a faulty working condition. This paper investigates the use of mathematical models to track these normal operating conditions, and the models themselves are trained using normal data to obtain them. For abnormal data, the state variables of the model deviate from the normal distribution and the process is determined to be abnormal. However, the method in this paper will only monitor the abnormalities but not eliminate them, and the job of eliminating them is left to the fault diagnosis.

After analyzing the data characteristics of the ternary cathode material preparation industrial system, it is found that the production data has problems such as noise interference, dynamics, high dimension, and multi-modality [26]. First, in the sintering preparation process, the uncertainty of the production process such as the aging of the silicon carbide rod and the fluctuation of the exhaust pipe flow causes the acquisition device to be randomly interfered with by factors such as random errors and human errors. Secondly, due to the mutual coupling between the reactions of the device before and after, the samples at the current sampling time are affected by the state of the previous time and show strong dynamic characteristics. Third, because the sintering process has the characteristics of a long process and large delay, the sensors installed in the system will collect numerous variables to obtain the sintering state, resulting in a high dimension of data that is involved. Fourth, the chemical reaction that occurs during the sintering process is particularly sensitive to the temperature of the external environment [27]. When there are periodic disturbances in the external environment, such as seasonal changes, the system will adjust the operating parameters to compensate for the influence of external disturbances. The system has a variety of stable working states, that is, the distribution of the collected data will change periodically with the change in the external environment. To sum up, the sintering process data of ternary cathode material exhibit randomness, dynamics, high latitude, and multi-modal characteristics. Traditional monitoring and monitoring methods cannot overcome these characteristics to satisfy the monitoring effect required by the industry.

## 3. Factor Dynamic Autoregressive Latent Variable Model

In view of the multimode characteristics of complex production process data, this section proposes a factor dynamic autoregressive latent variable model (FDALM) from the data perspective. The technique generalizes a single higher-order linear dynamic system to a multimodal-adaptive process for modeling multimodal features. Subsequently, an improved EM algorithm is proposed for model parameter identification to ensure the inter-class distances of different models.

### 3.1. Model Structure

**Figure 3:**
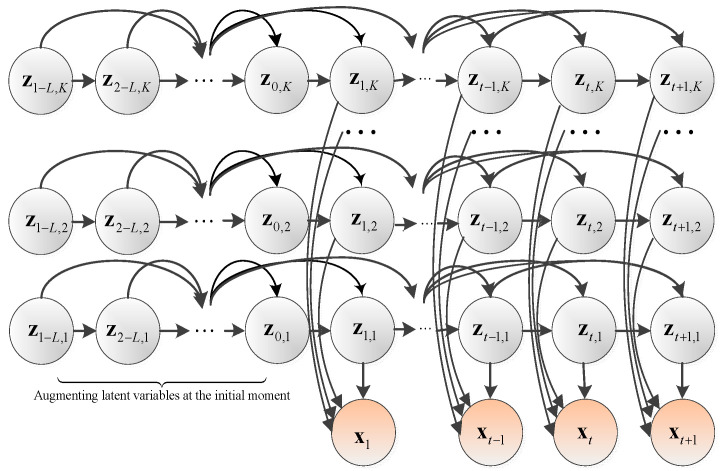
Topology of FDALM.

Most of the traditional data modeling methods are based on the assumption of a single-mode and a static process, which makes the monitoring method not capable of multimode tracking, resulting in false alarms, misdiagnoses, and even failures in monitoring results. Therefore. for multimode process data modeling, the modal representation is very important. The division between different modes requires a comprehensive trade-off between the dispersion and ductility of each mode, that is, the modes cannot overlap excessively, and the description of the modes is effective and sufficient. In order to make full use of the effective transition information between data series, the adopted factor modeling method is used to extend the single-modal DALM to obtain the modeling method of factor dynamic autoregressive latent variable model (FDALM). The probability topology of the factor dynamic autoregressive latent variable model is shown in Figure 3.

Multi-modal processes are also generally dynamic. On the one hand, when the system is stable in a certain mode, the states at different times will interact with each other. In addition, the switching between different modes is also a dynamic process. Therefore, whether it is data modeling or modal partitioning, dynamic information cannot be ignored. It can be seen from the above topology diagram that when there are *K* modes in the system, each mode is simulated by a high-order Markov state chain, which fully simulates the dynamic evolution law of a single mode. At the same time, *K* modalities exist at each moment, so the output observation variable is weighted with a weight coefficient, and each state is weighted with a factor weight *P*(*k*) to form the observation variable **x** at the current time. The mathematical structure of FDALM is shown in Equation (1).

(1) $$\begin{array}{l} z_{t,k}=A_{k}h_{t-1,k}+\eta_{k}^{z}\\ x_{t,k}=B_{k}z_{t,k}+\eta_{k}^{x}\\ x_{t}=\sum_{k=1}^{K}P(k)x_{t,k} \end{array},$$

where $z_{t,k}\in R^{d}$ represents the latent variable describing the system at time t in mode *k*, $h_{t,k}$ represents the augmented latent variable composed of latent variables in mode k at time t and the first *L* − 1 moments, namely $h_{t-1,k}={\left[\begin{array}{cccc} z_{t-1,k}{}^{T} & z_{t-2,k}{}^{T} & \cdots & z_{t-L,k}{}^{T} \end{array}\right]}^{T}\in R^{dL}$, where *L* is the dynamic order. Then identify the order coefficients learned by the algorithm. $x_{t,k}\in R^{v}$ represents the process observation variable composed of the process variable and the quality variable in the mode *k* at time *t*, and d and v represent the dimensions of the latent variable and the observed variable, respectively. $A_{k}\in R^{d\times dL}$ represents the state transition matrix from the augmented latent variable $h_{t-1,k}$ to the latent variable $z_{t,k}$ at the current moment. $B_{k}\in R^{v\times d}$ represents the divergence matrix of the latent variable $z_{t,k}$ to the observed variable $x_{t,k}$. $\eta_{k}^{z}\in R^{d}$ and $\eta_{k}^{x}\in R^{v}$ are the Gaussian noises of the latent variable and observed variable in mode *k*, respectively. P(k) denotes the prior probability of the *k*th modality, $P(k|x_{i})$ denotes the posterior probability of the *k*th modality under x_i_ conditions; $x_{t}$ denotes the data collected at moment *t*, i.e., for the roller kiln temperature variable of this object. Assuming that the noises are independent of each other, the distributions obeyed are $\eta_{k}^{z}∼N(0,\Sigma_{z,k})$ and $\eta_{k}^{x}∼N(0,\Sigma_{x,k})$, respectively. The parameter Θ of the whole factor dynamic autoregressive latent variable model is shown in Equation (2).

(2) $$\Theta =\left\{A_{k},B_{k},\mu_{\pi }{}_{,k},\Sigma_{\pi ,k},\Sigma_{z,k},\Sigma_{x,k},P(k)|k=1,2,\cdots ,K\right\}.$$

Among them, $\mu_{\pi }{}_{,k}$ and $\Sigma_{\pi ,k}$ are the mean and variance of the Gaussian distribution of the augmented latent variable $h_{0,k}$ at the initial time, respectively.

### 3.2. FDALM Parameter Identification

Compared with DALM parameter identification, the factor modeling method not only needs to ensure the maximum likelihood function of a single model, but also needs to ensure that different model structures do not overlap excessively. Due to the existence of two types of unobservable variables in FDALM structure, which are the latent variables of the model and the factor coefficients P(k), the traditional maximum likelihood function cannot be used. In this section, an improved EM algorithm [28] is proposed to identify the parameters, which shortens the learning time and ensures the inter-class distance. Similar to each single DALM model, in the *k*th sub-DALM, the conditional probability distribution of the latent variable $z_{t,k}$ and the observed variable $x_{t,k}$ is shown in Equation (3).

(3) $$\begin{array}{l} h_{0,k}∼N(\mu_{\pi ,k},\Sigma_{\pi ,k})\\ z_{t,k}|h_{t-1,k}∼N(A_{k}h_{t-1,k},\Sigma_{z,k})\\ x_{t,k}|z_{t,k}∼N(B_{k}z_{t,k},\Sigma_{x,k}) \end{array}$$

The parameters that the model needs to identify are $\Theta =\{A_{k},B_{k},\mu_{\pi }{}_{,k},\Sigma_{\pi ,k},\Sigma_{z,k},\Sigma_{x,k},$ P(k)|k=1,2,⋯,K}, where $A_{k}=\left[A_{1,k},A_{2,k},\cdots ,A_{L,k}\right]$. The goal of identification is to obtain the optimal parameter set of the model by giving the observation data matrix data set $x_{1:T}={\left[x_{1},x_{2},\cdots ,x_{T}\right]}^{T}\in R^{T\times v}$, where *T* is the number of samples in the training data set. The optimal solution of the model parameters is maximized by solving the log-likelihood function of the model, as shown in Equation (4).

(4) $$\Theta^{opt}\;=\;\arg\underset{\Theta }{\max}L\left(\Theta \right)=\arg\;\underset{\Theta }{\max}In\left(x_{1:T}|\Theta \right)$$

In the E step of the EM algorithm, the objective cost function is first constructed, that is, the mathematical expectation of the conditional posterior probability $P(z_{1-L:T},k|x_{1:T},\Theta^{old})$ of the complete log-likelihood function $InP(x_{1:T},z_{1-L:T}|\Theta )$ about the latent variable is calculated, as shown in Equation (5).

(5) $$\begin{array}{l} Q\left(\Theta |\Theta^{old}\right)=E\left(L\left(\Theta \right)\right)=E\left\{InP\left(x_{1:T}|\Theta^{old}\right)\right\}\\ \;\;=\sum_{k=1}^{K}E_{P\left(z_{1-L:T},k|x_{1:T},\Theta^{old}\right)}\left[In\left(x_{1:T},z_{1-L:T},k|\Theta^{old}\right)\right]\\ \;\;=\sum_{k=1}^{K}P\left(k|x_{1:T}\right)E_{P\left(z_{1-L:T}|x_{1:T},k,\Theta^{old}\right)}\left[In\left(x_{1:T},z_{1-L:T},k|\Theta^{old}\right)\right] \end{array}.$$

From the Bayesian topology knowledge, combined with the DALM topology, Equation (5) is expanded.

(6) $$\begin{array}{l} InP(x_{1:T},z_{1-L:T},k)=In\left\{P(k)P\left(x_{1:T},z_{1-L:T}|k\right)\right\}\\ =\mathrm{In}P(k)+InP(h_{0,k}|k)+\sum_{t=1}^{T}\left\{InP(z_{t,k}|h_{t-1,k})+InP(x_{t}|z_{t,k},k)\right\} \end{array}.$$

Among them, P(k) is the factor coefficient and obeys $\sum_{k=1}^{K}P\left(k\right)=1$. For the sake of writing convenience, this section will denote the conditional expectation $E_{z_{1-L:T,k}|x_{1:T},k,\Theta^{old}}\left\{f\left(x_{t},z_{t,k}\right)\right\}$ on the latent variable as $E_{Z}\left\{f\left(x_{t},z_{t,k}\right)\right\}$. The mathematical analysis formula of the objective cost function expansion is obtained as Equation (7).

(7) $$\begin{array}{l} Q\left(\Theta |\Theta^{old}\right)=\sum_{k=1}^{K}P\left(k|x_{1:T}\right)\times InP(k)\\ -\sum_{k=1}^{K}\frac{P\left(k|x_{1:T}\right)}{2}\times \left\{In\left|\Sigma_{\pi ,k}\right|+E_{Z}\left(h_{0,k}\Sigma_{\pi ,k}^{-1}h_{0,k}^{T}\right)-2E_{Z}\left(h_{0,k}\right)\Sigma_{\pi ,k}^{-1}\mu_{\pi ,k}+\mu_{\pi ,k}^{T}\Sigma_{\pi ,k}^{-1}\mu_{\pi ,k}\right\}\\ -\sum_{t=1}^{T}\sum_{k=1}^{K}\frac{P\left(k|x_{t}\right)}{2}\times \left\{\begin{array}{l} In\left|\Sigma_{z,k}\right|+E_{Z}\left(z_{t,k}^{T}\Sigma_{z,k}^{-1}z_{t,k}\right)-2E_{Z}\left(h_{t-1,k}A_{k}^{T}\Sigma_{z,k}^{-1}z_{t,k}\right)\\ +E_{Z}\left(h_{t-1,k}A_{k}^{T}\Sigma_{z,k}^{-1}A_{k}h_{t-1,k}^{T}\right) \end{array}\right\}\\ -\sum_{t=1}^{T}\sum_{k=1}^{K}\frac{P\left(k|x_{t}\right)}{2}\times \left\{\begin{array}{l} In|\Sigma_{x,k}|+x_{t}^{T}\Sigma_{x,k}^{-1}x_{t}-2E_{Z}\left(z_{t,k}^{T}\right)B_{k}^{T}\Sigma_{x,k}^{-1}x_{t,k}\\ +E_{Z}\left(z_{t,k}^{T}B_{k}^{T}\Sigma_{x,k}^{-1}B_{k}z_{t,k}\right) \end{array}\right\}+cons \end{array}.$$

In the E step of the EM algorithm, given the iterative parameter $\Theta^{old}$ of the previous step, the mathematical expectation of the conditional probability of two types of latent variables is obtained. One is the conditional expectation of the latent variable, namely $E_{Z}\left(z_{t,k}\right)$, $E_{z}\left(z_{t,k}z_{t,k}^{T}\right)$, and $E_{Z}\left(z_{t,k}z_{t-i,k}^{T}\right)$, where t=0,1,⋯,T, i=1,2,⋯,L. The derivation process is similar to the E-step of the traditional DALM model, and will not be introduced due to limited space. The final result is given as Equation (8).

(8) $$\left\{ \begin{array}{l} E_{Z}\left(z_{t,k}\right)=E\left(z_{t,k}|x_{1:T},\Theta^{old}\right)=m_{t,k}^{1}\\ E_{Z}\left(z_{t,k}z_{t,k}^{T}\right)=E\left(z_{t,k}z_{t,k}^{T}|x_{1:T},\Theta^{old}\right)=M_{t,k}^{11}+m_{t,k}^{1}{\left(m_{t,k}^{1}\right)}^{T}\\ E_{Z}\left(z_{t,k}z_{t-i,k}^{T}\right)=E\left(z_{t,k}z_{t-i,k}^{T}|x_{1:T},\Theta^{old}\right)=M_{t,k}^{1(i+1)}+m_{t,k}^{1}{\left(m_{t,k}^{\left(i+1\right)}\right)}^{T}\\ E_{Z}\left(z_{t,k}z_{t-L,k}^{T}\right)=E\left(z_{t,k}z_{t-L,k}^{T}|x_{1:T},\Theta^{old}\right)=\sum_{i=1}^{L}A_{i,k}\left(M_{t-1,k}^{iL}+m_{t-1,k}^{i}{\left(m_{t-1,k}^{L}\right)}^{T}\right) \end{array}\right..$$

Among them, $m_{t,k}$ and $M_{t,k}$ are the mean and variance of the posterior probability distribution of the *k*th sub-modal latent variable $h_{t,k}$ with respect to the observation sequence $x_{1:T}$, respectively. Another type of latent variable is the posterior probability $P\left(k|x_{t}\right)$ of factor *k*, where t=0,1,⋯,T. The mathematical solution is shown in Equation (9).

(9) $$P\left(k|x_{t}\right)=\frac{P\left(x_{t}|k\right)P\left(k\right)}{P\left(x_{t}\right)}.$$

Among them, P(k) is the factor coefficient of the previous iteration. In the M step of the EM algorithm, the full log-likelihood expectation function $Q\left(\Theta |\Theta^{old}\right)$ is maximized by using the latent variable expectation and the posterior probability of the factor obtained in the E step, and a new iterative parameter set $\Theta^{new}$ is obtained. First, we maximize the objective cost function $Q\left(\Theta |\Theta^{old}\right)$, as shown in Equation (10).

(10) $$\Theta^{new}=\arg\underset{\Theta }{\max}Q\left(\Theta |\Theta_{old}\right).$$

Let the cost function $Q\left(\Theta |\Theta^{old}\right)$ be derived separately from the model parameters and equal to zero, as shown in Equation (11).

(11) $$\frac{\partial Q(\Theta |\Theta_{old})}{\partial \Theta }=0.$$

The detailed update process is shown as the following.

(12) $$\begin{array}{l} \frac{\partial \left\{Q\left(\Theta |\Theta^{old}\right)\right\}}{\partial \mu_{\pi ,k}}=0\Rightarrow \frac{\partial \left\{2E_{Z}\left(h_{0,k}\right)\Sigma_{\pi ,k}^{-1}\mu_{\pi ,k}+\mu_{\pi ,k}^{T}\Sigma_{\pi ,k}^{-1}\mu_{\pi ,k}\right\}}{\partial \mu_{\pi ,k}}=0\\ \Rightarrow -2\Sigma_{\pi ,k}^{-1}E_{Z}\left(h_{0,k}\right)+2\Sigma_{\pi ,k}^{-1}\mu_{\pi ,k}=0\Rightarrow \mu_{\pi ,k}^{new}\;=\;E_{z}\left(h_{0,k}\right) \end{array},$$

(13) $$\begin{array}{l} \frac{\partial \left\{Q\left(\Theta |\Theta^{old}\right)\right\}}{\partial \Sigma_{\pi ,k}}=0\\ \Rightarrow \frac{\partial \left\{In\left|\Sigma_{\pi ,k}\right|+E_{Z}\left(h_{0,k}\Sigma_{\pi ,k}^{-1}h_{0,k}^{T}\right)-2E_{Z}\left(h_{0,k}\right)\Sigma_{\pi ,k}^{-1}\mu_{\pi ,k}+\mu_{\pi ,k}^{T}\Sigma_{\pi ,k}^{-1}\mu_{\pi ,k}\right\}}{\partial \Sigma_{\pi ,k}}=0\\ \Rightarrow \Sigma_{\pi ,k}^{-1}-\Sigma_{\pi ,k}^{-1}E_{z}\left(h_{0,k}h_{0,k}^{T}\right)\Sigma_{\pi ,k}^{-1}+\Sigma_{\pi ,k}^{-1}E_{z}\left(h_{0,k}\right)E\left(h_{0,k}^{T}\right)\Sigma_{\pi ,k}^{-1}=0\\ \Rightarrow \Sigma_{\pi ,k}^{new}=E_{z}\left(h_{0,k}h_{0,k}^{T}\right)-E_{z}\left(h_{0,k}\right)E\left(h_{0,k}^{T}\right) \end{array},$$

(14) $$\begin{array}{l} \frac{\partial \left\{Q\left(\Theta |\Theta^{old}\right)\right\}}{\partial A_{k}}=0\\ \Rightarrow -\sum_{t=1}^{T}P\left(k|x_{t}\right)\Sigma_{z,k}^{-1}E_{z}\left(z_{t,k}h_{t-1,k}^{T}\right)+\sum_{t=1}^{T}P\left(k|x_{t}\right)\Sigma_{z,k}^{-1}A_{k}E_{z}\left(h_{t-1,k}h_{t-1,k}^{T}\right)=0\\ \Rightarrow A_{k}^{new}=\sum_{t=1}^{T}P\left(k|x_{t}\right)E_{z}\left(z_{t,k}h_{t-1,k}^{T}\right){\left[\sum_{t=1}^{T}P\left(k|x_{t}\right)E_{z}\left(h_{t-1,k}h_{t-1,k}^{T}\right)\right]}^{-1} \end{array},$$

(15) $$\begin{array}{l} \frac{\partial \left\{Q\left(\Theta |\Theta^{old}\right)\right\}}{\partial B_{k}}=0\\ \Rightarrow \frac{\partial \left\{\sum_{t=1}^{T}P\left(k|x_{t}\right)\left[-2E_{Z}\left(z_{t,k}^{T}\right)B_{k}^{T}\Sigma_{x,k}^{-1}x_{t,k}+E_{Z}\left(z_{t,k}^{T}B_{k}^{T}\Sigma_{x,k}^{-1}B_{k}z_{t,k}\right)\right]\right\}}{\partial B_{k}}=0\\ \Rightarrow -\sum_{t=1}^{T}P\left(k|x_{t}\right)\Sigma_{x,k}^{-1}x_{t,k}E_{z}\left(z_{t,k}^{T}\right)+\sum_{t=1}^{T}P\left(k|x_{t}\right)\Sigma_{x,k}^{-1}B_{k}E_{z}\left(z_{i,k}z_{t,k}^{T}\right)=0\\ \Rightarrow B_{k}^{new}=\sum_{t=1}^{T}P\left(k|x_{t}\right)x_{t}E_{z}\left(z_{t,k}^{T}\right){\left[\sum_{t=1}^{T}P\left(k|x_{t}\right)E_{z}\left(z_{i,k}z_{t,k}^{T}\right)\right]}^{-1} \end{array},$$

(16) $$\begin{array}{l} \frac{\partial \left\{Q\left(\Theta |\Theta^{old}\right)\right\}}{\partial \Sigma_{z,k}}=0\\ \Rightarrow \sum_{t=1}^{T}P\left(k|x_{t}\right)\left[\begin{array}{l} \Sigma_{z,k}^{-1}-\Sigma_{z,k}^{-1}E_{z}\left(z_{t,k}z_{t,k}^{T}\right)+\Sigma_{z,k}^{-1}A_{k}E_{z}\left(h_{t-1,k}z_{t,k}^{T}\right)A_{k}^{T}\Sigma_{z,k}^{-1}\\ +\Sigma_{z,k}^{-1}A_{k}E_{z}\left(z_{t,k}z_{t,k}^{T}\right)A_{k}^{T}\Sigma_{z,k}^{-1} \end{array}\right]=0\\ \Rightarrow \Sigma_{z,k}^{new}=\frac{\sum_{t=1}^{T}P\left(k|x_{t}\right)\left[E_{z}\left(z_{t,k}z_{t,k}^{T}\right)-A_{k}^{new}E_{z}\left(h_{t-1,k}z_{t,k}^{T}\right){\left(A_{k}^{new}\right)}^{T}+A_{k}^{new}E_{z}\left(z_{t,k}z_{t,k}^{T}\right){\left(A_{k}^{new}\right)}^{T}\right]}{\sum_{t=1}^{T}P\left(k|x_{t}\right)} \end{array},$$

(17) $$\begin{array}{l} \frac{\partial \left\{Q\left(\Theta |\Theta^{old}\right)\right\}}{\partial \Sigma_{z,k}}=0\\ \Rightarrow \sum_{t=1}^{T}P\left(k|x_{t}\right)\left[\begin{array}{l} \Sigma_{x,k}^{-1}-\Sigma_{x,k}^{-1}x_{t}x_{t}^{T}\Sigma_{x,k}^{-1}+\Sigma_{x,k}^{-1}B_{k}E_{z}\left(h_{t,k}\right)x_{t}^{T}\Sigma_{x,k}^{-1}\\ +\Sigma_{z,k}^{-1}B_{k}E_{z}\left(z_{t,k}z_{t,k}{}^{T}\right)B_{k}^{T}\Sigma_{z,k}^{-1} \end{array}\right]=0\\ \Rightarrow \Sigma_{x,k}^{new}=\frac{\sum_{t=1}^{T}P\left(k|x_{t}\right)\left[x_{t}x_{t}^{T}-2B_{k}^{new}E_{z}\left(h_{t,k}\right)x_{t}^{T}+B_{k}^{new}E_{z}\left(z_{t,k}z_{t,k}{}^{T}\right){\left(B_{k}^{new}\right)}^{T}\right]}{\sum_{t=1}^{T}P\left(k|x_{t}\right)} \end{array}.$$

In order to update the factor coefficient P(k), since the factor involves more constraints, an optimization objective needs to be constructed separately for it. First, all terms related to P(k) are separated and represented as Equation (18).

(18) $$g(k)=\sum_{t=1}^{T}\sum_{k=1}^{K}P\left(k|x_{t}\right)InP\left(k\right).$$

Due to the existence of constraints $\sum_{k=1}^{K}P(k)=1$, the Lagrange multiplier is introduced and constructed into a Lagrange function form.

(19) $$f\left(k\right)=g\left(k\right)+\lambda \left(\sum_{k=1}^{K}P\left(k\right)-1\right),$$

(20) $$\sum_{t=1}^{T}P(k|x_{t})+\lambda P\left(k\right)=0\Rightarrow P\left(k\right)=-\frac{\sum_{t=1}^{T}P(k|x_{t})}{\lambda }.$$

We accumulate the *k* terms (for *k*) on both sides of Equations (19) and (20).

(21) $$\begin{array}{l} \sum_{k=1}^{K}P(k)=-\frac{\sum_{t=1}^{T}\sum_{k=1}^{K}P\left(k|x_{t}\right)}{\lambda }\\ \sum_{k=1}^{K}P(k|x_{t})=1\\ \sum_{k=1}^{K}P\left(k\right)=1 \end{array}\}\Rightarrow \lambda =-T.$$

We substitute the result back into Equation (20) to obtain the result of the factor coefficient.

(22) $$P(k)=\frac{1}{T}\sum_{t=1}^{T}P(k|x_{t}).$$

We update the obtained parameter set $\Theta^{new}=\{A_{k}^{new},B_{k}^{new},\mu_{\pi ,k}^{new},\Sigma_{\pi ,k}^{new},\Sigma_{z,k}^{new},\Sigma_{x,k}^{new},P(k)$ |k=1,2,⋯,K}, and iterate E and M steps to make the parameter matrix converge, that is, to satisfy Equation (23).

(23) $$\left|\right|\Theta^{old}-\Theta^{new}\left|\right|\leq \sigma .$$

**Figure 4:**
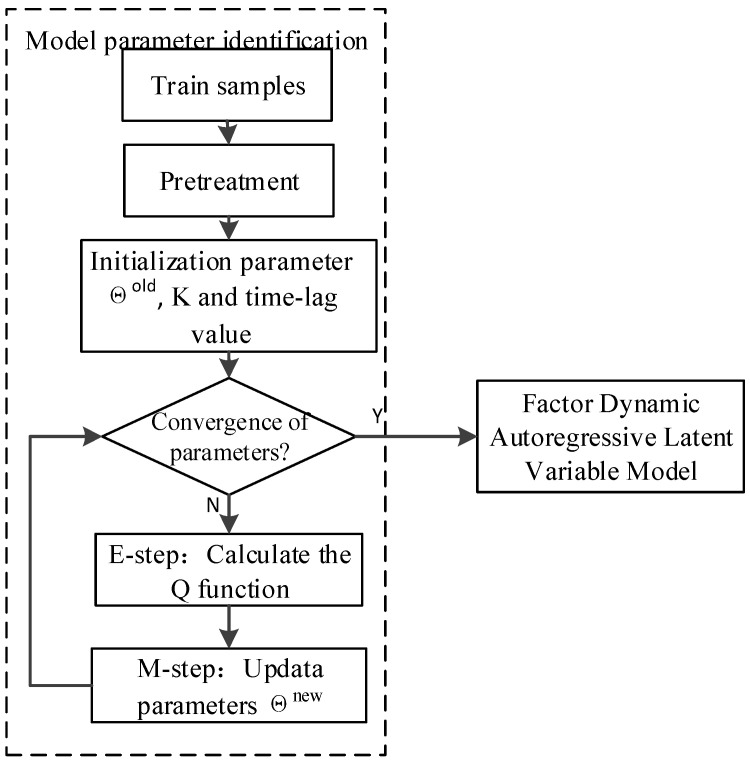
The flow chart of model training.

The optimal parameter set Θ is obtained. At this point, the parameter learning process of FDALM is over. The model is generated by training the data under normal operating conditions. Firstly, the normal data is pre-processed by normalization. Then the EM algorithm identifies the parameters. Finally, we obtain the trained FDALM. The flow chart of model training can be seen in Figure 4.

## 4. FDALM-Based Multimodal Process Monitoring Method

In order to avoid the segmentation of the process data leading to the destruction of the dynamic information in the sequence, it is necessary to make full use of the output of each modal factor in FDALM. However, the output of each factor mode is a representation of probability. If the construction statistics of each sub-mode are monitored separately, local false positives are prone to occur, and the overall monitoring effect cannot be achieved. Therefore, this chapter focuses on the use of Bayesian inference technology to fuse different factor modalities into the posterior failure probability of the final sample, so as to monitor the fluctuation of the whole process state in real time.

For a trained FDALM, the latent variable is the core force driving the operation of the system. Therefore, this variable contains important key state information of the modality. Although the latent variables of each sub-mode can establish the corresponding $T_{t,k}^{2}$ statistic.

(24) $$T_{t,k}^{2}=E{\left(z_{t}{}^{q}|x_{1:t}{}^{q}\right)}^{T}cov{\left(Z_{k}\right)}^{-1}E\left(z_{t}{}^{q}|x_{1:t}{}^{q}\right).$$

$Z_{k}$ is the set of latent variables in the *k*th mode in the training set, and $cov(Z_{k})$ is the covariance of $Z_{k}$. However, different sub-models have their own control limits, and it is difficult to achieve the overall monitoring effect. In order to make full use of the sample posterior latent variable output in each mode, the monitoring results of each sub-mode are fused into the sample posterior failure probability by means of Bayesian inference method. First, we construct the probability of failure of the observation sample in the *k*th mode.

(25) $$\begin{array}{l} P_{T^{2}}^{k}\left(F|x_{t}^{q}\right)=\frac{P_{T^{2}}^{k}\left(x_{t}^{q}|F\right)P_{T^{2}}^{k}\left(F\right)}{P_{T^{2}}^{k}\left(x_{t}^{q}\right)}\\ P_{T^{2}}^{k}\left(x_{t}^{q}\right)=P_{T^{2}}^{k}\left(x_{t}^{q}|N\right)P_{T^{2}}^{k}\left(N\right)+P_{T^{2}}^{k}\left(x_{t}^{q}|F\right)P_{T^{2}}^{k}\left(F\right) \end{array}.$$

Among them, $P_{T^{2}}^{k}\left(F\right)$ and $P_{T^{2}}^{k}\left(N\right)$ represent the prior probability of abnormal and normal process data, which can be skillfully combined with the significance level α.

(26) $$\begin{array}{l} P_{T^{2}}^{k}\left(F\right)=1-\alpha \\ P_{T^{2}}^{k}\left(N\right)=\alpha \end{array}.$$

Among them, α is the significance level, and the actual size is the balance between false positives and false negatives. In order to obtain the failure probability $P_{T^{2}}^{k}\left(F|x_{t}^{q}\right)$ of a new data sample, it is necessary to construct the conditional probability of the observed samples under normal and abnormal conditions. In this section, they are defined as Equation (27).

(27) $$\begin{array}{l} P_{T^{2}}^{k}\left(x_{t}|N\right)=\exp\left\{-\frac{T_{t,k}^{2}}{T_{\lim,k}^{2}}\right\}\\ P_{T^{2}}^{k}\left(x_{t}|F\right)=\exp\left\{-\frac{T_{\lim,k}^{2}}{T_{t,k}^{2}}\right\} \end{array}.$$

Among them, $T_{\lim,k}^{2}$ is the control limit of each mode, and its value is uniquely determined by the degree of freedom d of the sub-model and the significance level α; $T_{t,k}^{2}$ is the $T^{2}$ statistic of the *k*th sub-mode, and the calculation method is shown in Equation (24). After the probability of failure of each local model is determined in the observation data, Bayesian inference technology is used to further fuse the probability of failure of the sample in each sub-mode to obtain the posterior failure probability $T_{t,final}^{2}$ of the sample.

(28) $$T_{t,final}^{2}=\sum_{k=1}^{K}P\left(k|x_{t}\right)P_{T^{2}}^{k}\left(F|x_{t}\right).$$

By comparing the posterior failure probability with the significance level α, it is judged whether the system fails. The judgment logic is shown in Equation (29).

(29) $$\left\{ \begin{array}{l} T_{t,final}^{2}\leq \alpha ,\;\;\;\;\;\mathrm{normal}\\ T_{t,final}^{2}>\alpha ,\;\;\;\;\;\mathrm{abnormal} \end{array}\right..$$

The flow chart of process data modeling and monitoring is shown in Figure 4. Since the detection by constructing chi-square statistics for a single mode will result in a local false alarm, to improve the overall monitoring effect, this paper calculates the posterior failure probability of the sample in a certain mode. We then obtain the posterior failure probability of the sample by using Bayesian fusion methods. If the actual solitary probability of the sample exceeds the threshold, the monitoring is abnormal, and the control threshold is the average value between the failure rate and false alarm rate, which is generally taken as 0.05, i.e., tolerating 5% of false alarms.

Further, the main steps of the FDALM-based multimodal process monitoring method are shown in Algorithm 1:
**Algorithm 1: Steps of an FDALM-based Process Monitoring Method****Input:** In order to train the model parameters, the observation variables are selected from the process variables and quality variables, and the sampling sequence of *T* consecutive moments is selected as the training data set, denoted as $x_{1:T}$; in order to test the monitoring performance, the sampling sequence of N consecutive moments is selected online as the Test data set, denoted as $x_{1:N}$. **Step 1:** The training and testing data sets $\left\{x_{1:T},x_{1:N}\right\}$ are preprocessed by a normalization method. **Step 2:** Use the dynamic order identification algorithm and clustering algorithm to identify the order coefficient L and factor K of the model, determine the structure of FDALM, and initialize the model parameters $\Theta^{old}$. **Step 3:** Identification of FDALM Parameters Using Improved EM Algorithm. **Step 4:** Determine the control threshold $T_{k,\lim}^{2}$ and significance level α of the sub-model, calculate the latent variable distribution of each observation sample under each sub-model online, and use the Bayesian inference technology to fuse the output of each sub-mode into the sample posterior failure probability $T_{t,final}^{2}$. **Step 5:** Compare $T_{t,final}^{2}$ with the significance level α to judge the state of the process. **Output:** Output system operating status.

## 5. Application Research on the Preparation Process of Ternary Cathode Material

### 5.1. Model Building

To establish a reasonable mathematical model for the preparation process data, it is necessary to select the observed variables from the system process variables as the model input. Since the distribution of temperature field during sintering will have a significant impact on the performance of battery material, the specific manifestations are: over-burning will cause changes in material morphology and internal structure, and under-burning will not provide sufficient activation energy for chemical reactions. By analyzing the mechanism of the sintering process, the decomposition reaction in the heating stage is an endothermic process that requires sufficient and suitable heat. Under-burning will lead to low water removal efficiency and affect subsequent oxidation reactions; over-burning will lead to energy waste and increase production costs. Therefore, a reasonable temperature distribution is very important for the sintering process. By analyzing the actual production process data, the temperature sensors installed on the upper and lower sides of each temperature zone can indirectly reflect the temperature distribution of the sintering temperature zone. Reasonable temperature field distribution will help the production of high-quality products. At the same time, some process abnormal states are included in the product indicators. For example, the abnormality of the outlet replacement chamber will lead to an increase in the residual lithium content. In order to comprehensively monitor the common faults in the process, the temperature variables and residual lithium content of the entire temperature area are selected as the observation variables of the monitoring model.

In the actual production process, the main temperature anomalies are abnormal temperature increase/decrease and shutdown failures. Accidents such as the aging of the thermocouple and the fracture of the heating rod during the production process will cause the sintering temperature to rise abnormally. An abnormality such as the excessive current of the thermocouple and the large exhaust port can directly lead to an abnormal decrease of the temperature. The shutdown failure often occurs in the circuit short circuit, transmission jams, and other abnormalities. In order to verify the monitoring effect of the model under different faults, a total of 2200 consecutive time data were selected, and the sampling period was 30 min. The ternary cathode material roller kiln is generally stable during manufacturing and the temperature does not change much, so the sampling time in the plant is generally set as 30 min. All the data in this paper come from the industrial platform carried out in our laboratory. To simulate the continuity of the production process, all our faults are generated continuously, so the information at the time of continuous switching is retained to facilitate the ability of the model to model dynamically. It is not possible to put these different data segments together, because this would destroy the useful information between sequences, and it would be difficult to capture these dynamic changes when modeling dynamically, resulting in inaccurate modeling or a false alarm. The sequence data are collected under the same batch of battery material produced in the factory, and include the common process failures in the production process. In addition, the normalized mean and variance of the normalized data are used to normalize the training data and project their state distributions into a uniform space to facilitate the distinction between normal and abnormal states. The detailed physical description of this batch of data is shown in Table 1.

**Figure 5:**
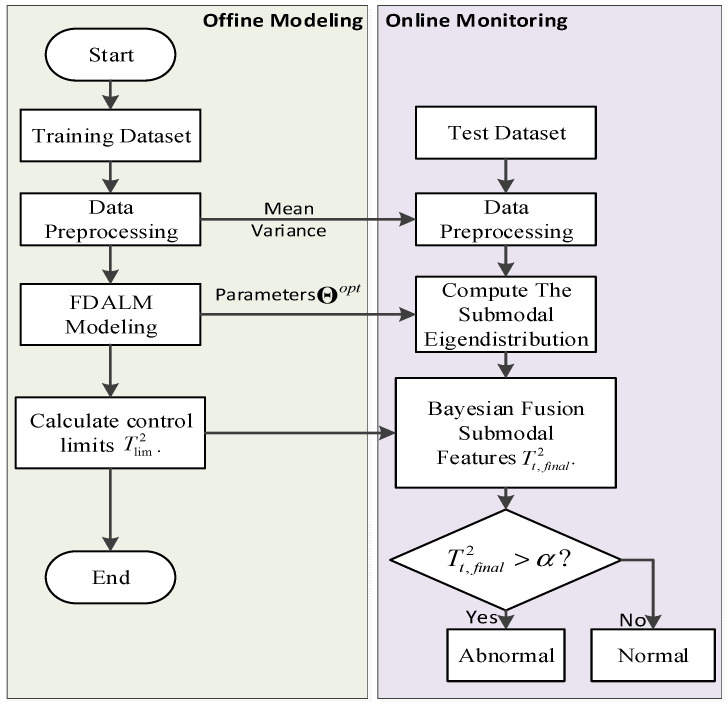
Flow chart of the process monitoring method based on FDALM.

The product quality of the ternary cathode material is particularly sensitive to the sintering temperature. From a macro perspective, it is affected by the fluctuation of battery raw material and ambient temperature. The environment temperature in summer is 30 °C and 10 °C in winter. In the actual manufacturing, to overcome fluctuation in the environment temperature, it is necessary to adjust the operating parameters, thus changing the optimal sintering system, so that there are two optimal sintering systems in different environments temperature. The process data is collected for multimodal characteristics. The process data is clustered by an improved affine propagation clustering algorithm [29], which can adaptively determine the optimal parameters of the algorithm, thereby obtaining the optimal factor *K* of FDALM. The data in the normal data interval, that is, the 1st–1000th data, is used to classify the data set. The result shows that the data is divided into two categories. In order to visualize the division effect, the data is subjected to PCA dimension reduction, and the contribution is 99%. The first two-dimensional variables of the data are visualized, as shown in Figure 5.

It can be seen from the visualization result that the classification algorithm divides the process data into two categories. The results fully demonstrate that the data obeys multiple distributions, so the assumption of a single distribution does not hold. Accurate monitoring of this process requires further consideration of the multimodal nature of the process data.

In order to establish a factor dynamic autoregressive latent variable model, the model structure parameters that need to be determined include order coefficient *L* and factor *K*. Among them, the order coefficient *L* is identified by the trend similarity algorithm *L* = 3; the physical interpretation of the factor *K* is the number of types of data division, which is obtained by the improved affine propagation clustering algorithm, *K* = 2. In order to verify the rationality of factor selection, this section designs experiments to establish FDALM under different factor *K* and compare its monitoring effect. The normal sample interval 1st~1200th is selected as the training data set, and the fault 1 data set is used to test the monitoring effect of the model. In order to evaluate the actual monitoring effect of the monitoring method, two statistics, False Alarm Rate (*FAR*) and Fault Detection Rate (*FDR*), are used.

(30) $$\begin{array}{l} FAR=\frac{N_{FAR}}{N_{n}}\\ FDR=\frac{N_{FDR}}{N_{f}}. \end{array}$$

Among them, $N_{FAR}$ represents the number of normal samples misclassified by the monitored method, and $N_{n}$ is the number of all normal samples. $N_{FDR}$ is the number of fault samples correctly detected by the monitoring method, and $N_{f}$ is the number of all fault samples. Table 2 shows the monitoring effect of the method proposed in this section under different factor *K*.

**Figure 6:**
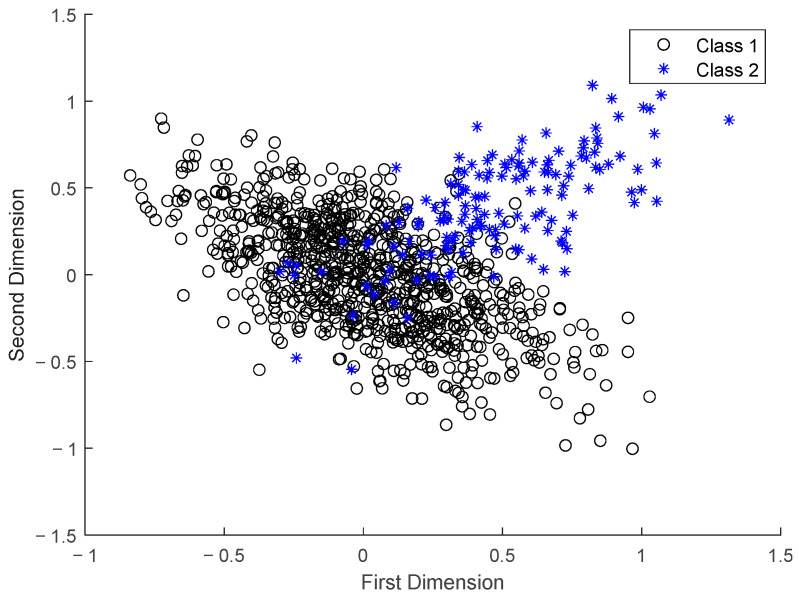
Process data classification diagram.

Table 2 shows the monitoring results indicators of FDALM under the factor *K* of (1, 2, 3). It can be seen that when factor *K* = 2, the method can obtain better monitoring results by combining the monitoring results and training costs. At the same time, Figure 6 shows the monitoring diagram of the monitoring method under different factors. It can be seen that choosing an appropriate *K* is helpful to smooth the monitoring results, and the monitoring results also verify the effectiveness of the classification algorithm. Finally, the factor *K* is selected as 2.

### 5.2. Process Monitoring

In order to compare the monitoring effect of the proposed monitoring method (FDALM) on dynamic multimode processes, two other monitoring methods are established for comparison in this section, which are representatives of static models: Probabilistic Partial Least Squares Regression (PPLSR) [30], and the representative of dynamic models: dynamic autoregressive latent variable model (DALM). Table 3 presents the *FAR* and *FDR* indicators of the monitoring results of the three monitoring methods in the data sets with different fault types.

**Figure 7:**
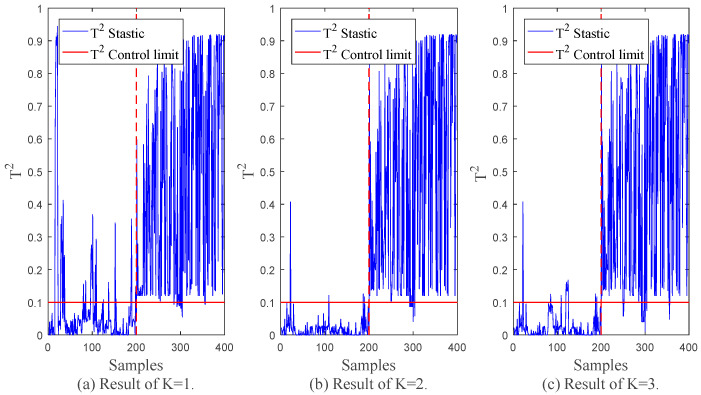
FDALM results at different *K*.

**Figure 8:**
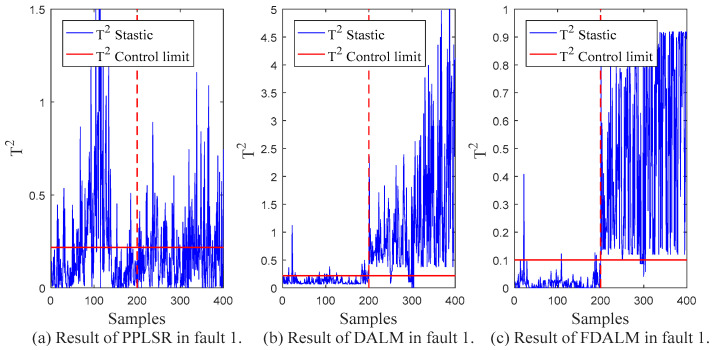
Monitoring results of fault 1.

**Figure 9:**
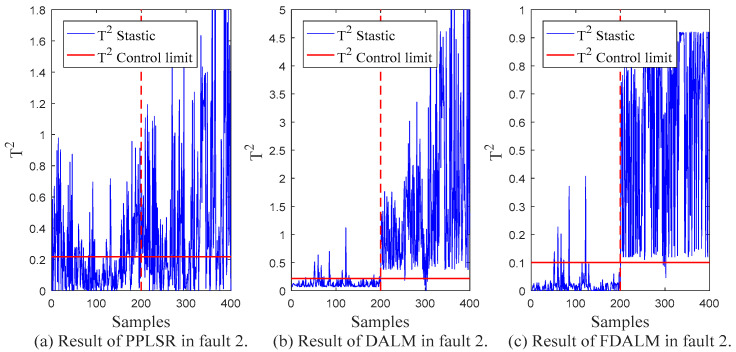
Monitoring results of fault 2.

**Figure 10:**
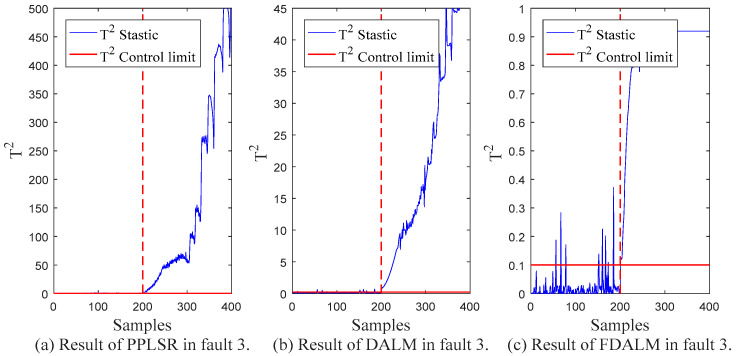
Monitoring results of fault 3.

From the final monitoring effect, FDALM is better than the DALM method, and DALM is better than the PPLSR method. Considering the structure of the model, the PPLSR model, as a static modeling method, only performs effective dimensionality reduction on the data set without considering the changes in the data sequence. Although DALM considers the dynamic information between sequences, it is difficult to adapt to monitoring of multimode processes; the FDALM model considers the dynamics and multimode characteristics of process data as a whole, so it is more comprehensive. In order to more intuitively show the monitoring performance at different times, Figure 7, Figure 8, Figure 9 and Figure 10 further present the monitoring results of the three models under different faults.

Compared with the traditional PPLSR, FDALM introduces both dynamic and multimodal information of the process data, which can better describe the dynamic changes and modal fluctuations of the sintering process. Therefore, the characterization of the process is more comprehensive. In addition, the advantages of FDLAM are also reflected in the modeling and fusion technology. First, the establishment of FDALM for process data can more accurately describe the dynamic and multimode characteristics of the process; it is prone to local false positives, and, finally, modeling sequence data does not lose inter-sequence information.

Compared with the single DALM monitoring method, the introduction of factor technology makes the modeling method suitable for multimode processes, which further improves the monitoring effect. The intuitive performance is that the parameters of the model in different modes of the process are different. Through factor weighting and Bayesian inference technology, the models established in different sub-modes can not only capture the modal information, but also establish local information for the fusion of the overall failure probability, so it can better follow the system dynamics and modal fluctuations. Compared with DALM, the overall fault false alarm rate and detection rate of the multimodal feature monitoring method are improved by 48.2% and 1.9%, respectively.

To sum up, the multimodal process monitoring method based on FDALM simultaneously solves the problems of dynamics, randomness, and multimodality of process data in the battery sintering process. It has a strong ability to describe complex production processes, and the monitoring results can play a better role in predicting the actual production process.

## 6. Conclusions

In this paper, a multimode process monitoring method based on FDALM was proposed. Firstly, in the technology of dynamic autoregressive latent variable model, with the help of the factor modeling method, DALM suitable for single mode was extended to multimode form, so that the model can consider the dynamic and multimode characteristics of process data at the same time. We introduced the use of the EM algorithm to learn the model parameters. Next, the failure probability of the sample under each sub-model was fused into the posterior failure probability of the sample by means of Bayesian inference technology to realize the process output of each factor model. Finally, the simulation results compared with other models show that the proposed monitoring method was able to track the modal fluctuations of the process.

An important issue in industrial process monitoring applications is the problem of multiple sampling rates. The method proposed in this paper assumes that the input and output data have the same sampling rate. If the sampling rate is inconsistent, some data is removed by down sampling. However, a more worthwhile approach is to combine semi-supervised learning methods, which can improve data utilization by training data on imbalanced input and output data. Another practical problem is the nonlinear relationships between process data, which are common in industrial processes. How to effectively deal with this issue deserves further research in the near future to make monitoring methods more applicable.

## Figures and Tables

**Table 1:** Process data descriptions.

Type	Data Description	Sampling Number
Normal	This interval is normal data	1st−1000th
Fault 1	1001st–1200th is normal data; 1201st–1400th is the abnormal temperature increase in the third temperature zone	1001st−1400th
Fault 2	1401st–1600th is normal data; 1601st–1800th is the abnormal temperature drop in the third temperature zone	1401st−1800th
Fault 3	1801st–2000th is normal data; 2001st–2200th is shutdown fault	1801st−2200th

**Table 2:** FDALM monitoring results for different *K*.

K	1	2	3
FAR	0.150	0.020	0.055
FDR	0.945	0.965	0.940

**Table 3:** Monitoring results for different faults.

Type	PPLSR		DALM		FDALM	
	*FAR*	*FDR*	*FAR*	*FDR*	*FAR*	*FDR*
Fault 1	0.240	0.120	0.120	0.925	0.020	0.965
Fault 2	0.100	0.350	0.090	0.970	0.045	0.985
Fault 3	0.315	0.980	0.085	1.000	0.040	1.000

## Data Availability

The data presented in this study are available upon request from the first author. The data are not publicly available due to intellectual property protection.
